# To a Tapeworm

**Published:** 1912-03

**Authors:** Bernard Wolff

**Affiliations:** Atlanta, Ga.


### TO A TAPEWORM.

Pale links of deviltry whose spectral length
Floats down the tube digestive, fold on fold.
Frail though they seem, yet show a giant's strength
When strong men bow themselves to loose their hold.

Ophidian head, square and tenaculate
Too small to harbor but a single thought—
To let the host all else evacuate
But cling forever to the gut it caught.

Those unrelenting hooks which never yield
When once upon the shrinking gut they seize
Know not defeat on any hard-fought field—
Can bring to earth a bellowing Hercules.

For naught the stricken bowels buck and strain,
Expulsive frenzies pass in sighs away.
They can not end the grim usurper's reign,
They can not break his intervillious sway.

High on the right a solemn sentry stands,
Old Hepar rears in might his curving pile,
And at the monster prone among the glands
Discharges broadsides in a squirt of bile.

On it in vain digestive torrents pour,
In vain the liver's well-directed squirt,
The stupid, pelted head but clings the more,
The body answers with a scornful flirt.

All men its mark or be they small or great,
The laborer delving in the stifling sewer,
The sovereign ruler of the Church or State,
Mayhap a Nell Gwinn or a Pompadour.

The flesh it melts, the ardent blood it cools,
The maiden's cheek grows wan, 'tis said for love,
But tell-tale links discovered in the stools,
Show trouble unromantic up above.

Insatiate glutton, those pale segments there
Know but one purpose, which is to be fed.
All things alike—it never knocks its fare
Be it a kingly dish or beggar's bread.

The fairest fruits are gathered for its feed,
The richest wines are cellared for its thirst,
The toil of many a one of Adam's seed
Goes to the nurture of this worm accurst.

Replete with food the languid cestode sleeps
Its rippling folds the chymous juices lave,
And o'er its length a soft contentment creeps
As undulates the peristaltic wave.

The days go by, the seasons come and wane,
The host reluctant waxes old, forsooth,
The parasite with never-ending chain
Keeps an immortal youth.

Nay, it also, with all that's born must pass,
Those pallid folds like banners must be furled,
What time a stagg'ring dose of filix mas
Shall thrust it gasping on the outer world.

Its reign is ended and its empire lost,
Its cysticerci scattered wide and far ,
This haughty monarch with vain hooklets crossed
Lies coiled forlornly in a Mason jar!

BERNARD WOLFF, M. D.

